# Cross-talk between EGF and BMP9 signalling pathways regulates the osteogenic differentiation of mesenchymal stem cells

**DOI:** 10.1111/jcmm.12097

**Published:** 2013-07-11

**Authors:** Xing Liu, Jiaqiang Qin, Qing Luo, Yang Bi, Gaohui Zhu, Wei Jiang, Stephanie H Kim, Mi Li, Yuxi Su, Guoxin Nan, Jing Cui, Wenwen Zhang, Ruidong Li, Xiang Chen, Yuhan Kong, Jiye Zhang, Jinhua Wang, Mary Rose Rogers, Hongyu Zhang, Wei Shui, Chen Zhao, Ning Wang, Xi Liang, Ningning Wu, Yunfeng He, Hue H Luu, Rex C Haydon, Lewis L Shi, Tingyu Li, Tong-Chuan He, Ming Li

**Affiliations:** a Stem Cell Biology and Therapy Laboratory of the Key Laboratory for Pediatrics designated by Chinese Ministry of Education and Chongqing Bureau of Education Department of Orthopaedic Surgery, The Children's Hospital of Chongqing Medical UniversityChongqing, China; b Molecular Oncology Laboratory Department of Orthopaedic Surgery, The University of Chicago Medical CenterChicago, IL, USA; c Ministry of Education Key Laboratory of Diagnostic Medicine Department of Laboratory Medicine and the Affiliated Hospitals of Chongqing Medical UniversityChongqing, China; d Department of Orthopaedic Surgery the Affiliated Tangdu Hospital, Fourth Military Medical UniversityXi'an, China; e School of Laboratory Medicine and the Affiliated Southwest Hospital, Third Military Medical UniversityChongqing, China

**Keywords:** BMP9 signalling, osteoblastic differentiation, EGF signalling, mesenchymal stem cells, osteogenic differentiation

## Abstract

Mesenchymal stem cells (MSCs) are multipotent progenitors, which give rise to several lineages, including bone, cartilage and fat. Epidermal growth factor (EGF) stimulates cell growth, proliferation and differentiation. EGF acts by binding with high affinity to epidermal growth factor receptor (EGFR) on the cell surface and stimulating the intrinsic protein tyrosine kinase activity of its receptor, which initiates a signal transduction cascade causing a variety of biochemical changes within the cell and regulating cell proliferation and differentiation. We have identified BMP9 as one of the most osteogenic BMPs in MSCs. In this study, we investigate if EGF signalling cross-talks with BMP9 and regulates BMP9-induced osteogenic differentiation. We find that EGF potentiates BMP9-induced early and late osteogenic markers of MSCs *in vitro*, which can be effectively blunted by EGFR inhibitors Gefitinib and Erlotinib or receptor tyrosine kinase inhibitors AG-1478 and AG-494 in a dose- and time-dependent manner. Furthermore, EGF significantly augments BMP9-induced bone formation in the cultured mouse foetal limb explants. *In vivo* stem cell implantation experiment reveals that exogenous expression of EGF in MSCs can effectively potentiate BMP9-induced ectopic bone formation, yielding larger and more mature bone masses. Interestingly, we find that, while EGF can induce BMP9 expression in MSCs, EGFR expression is directly up-regulated by BMP9 through Smad1/5/8 signalling pathway. Thus, the cross-talk between EGF and BMP9 signalling pathways in MSCs may underline their important roles in regulating osteogenic differentiation. Harnessing the synergy between BMP9 and EGF should be beneficial for enhancing osteogenesis in regenerative medicine.

## Introduction

Mesenchymal stem cells (MSCs) are multipotent progenitors and differentiate into osteogenic, chondrogenic, adipogenic and other lineages [1–5]. Although MSCs have been isolated from numerous tissues, one of the major sources in adults is the bone marrow stromal cells. Osteogenesis is a sequential cascade that recapitulates most of the cellular events occurring during embryonic skeletal development [6]. Bone is one of a few organs that retain the potential for regeneration and is the only tissue that can undergo continual remodelling throughout life. Bone morphogenetic proteins (BMPs) play an important role in development [4, 7, 8], stem cell proliferation and osteogenic differentiation [9, 10]. Bone morphogenetic proteins belong to the TGFβ [transforming growth factor (TGF)] superfamily and consist of at least 14 members in humans [4, 7, 8, 11, 12]. We previously analysed the osteogenic potential of the 14 types of BMPs and found that BMP9 is one of the most potent BMPs in inducing osteogenic differentiation of MSCs both *in vitro* and *in vivo* by regulating several important downstream targets during BMP9-induced osteoblast differentiation of MSCs [8, 13–21].

BMP9 (also known as growth differentiation factor 2, or GDF-2), originally identified in the developing mouse liver [22], may also play a role in regulating cholinergic phenotype [23], hepatic glucose and lipid metabolism [24], adipogenesis [25] and angiogenesis [26, 27]. Bone morphogenetic proteins initiate their signalling activity by binding to the heterodimeric complex of BMP type I and type II receptors [12]. We have recently demonstrated that BMP type I receptors ALK1 and ALK2 are essential for BMP9-induced osteogenic signalling in MSCs [28]. The activated receptor kinases phosphorylate Smads 1, 5 and/or 8, which in turn, regulate downstream targets in concert with co-activators during BMP9-induced osteoblast differentiation of MSCs [8, 13–20]. BMP9 is one of the least studied BMPs and its functional role in skeletal development remains to be fully understood.

It has been reported that epidermal growth factor (EGF) signalling may play an important role in endochondral bone formation and bone remodelling [29–31]. Epidermal growth factor is a key molecule in the regulation of cell growth and differentiation [30]. Earlier studies indicated that EGF administration at physiological doses induces distinct effects on endosteal and periosteal bone formation in a dose- and time-dependent manner [32, 33], although it was also reported that EGF exhibited biphasic effects on bone nodule formation in isolated rat calvaria cells *in vitro* [34]. Epidermal growth factor receptor (EGFR or ERBB1) is a transmembrane glycoprotein with intrinsic tyrosine kinase activity and activated by a family of seven peptide growth factors including EGF [31]. It is conceivable that the osteoinductive activity of BMP9 may be further regulated by cross-talking with other growth factors, such as EGF.

In this study, we investigate if EGF signalling cross-talks with BMP9 and regulates BMP9-induced osteogenic differentiation of MSCs. We show that EGF potentiates BMP9-induced early and late osteogenic markers of MSCs *in vitro*, which can be effectively blunted by EGFR inhibitors Gefitinib and Erlotinib or protein tyrosine kinase inhibitors AG-1478 and AG-494 in a dose-dependent manner. Furthermore, EGF significantly augments BMP9-induced endochondral bone formation in the cultured mouse foetal limb explants, which can be blocked by EGFR inhibitors. *In vivo* stem implantation experiments reveal that exogenous expression of EGF in MSCs effectively potentiates BMP9-induced ectopic bone formation, yielding larger and more mature trabecular bone masses. Mechanistically, EGF is shown to induce BMP9 expression in MSCs, whereas EGFR expression is directly up-regulated by BMP9 through Smad1/5/8 signalling pathway. Thus, the regulatory circuitry of EGF and BMP9 signalling pathways in MSCs may underline their important roles in regulating osteogenic differentiation. Harnessing the synergy between BMP9 and EGF may be beneficial for enhancing osteogenesis in regenerative medicine.

## Materials and methods

### Cell culture and chemicals

HEK293, C2C12 and C3H10T1/2 cells were from ATCC (Manassas, VA, USA). The reversibly immortalized mouse embryonic fibroblasts (iMEFs) were previously established [35]. Cell lines were maintained in the conditions as described [13, 15, 19, 36]. Recombinant human EGF (rhEGF) was purchased from Sigma-Aldrich (St. Louis, MO, USA). Epidermal growth factor receptor/tyrosine kinase inhibitors, including Gefitinib (aka, Iressa or ZD1839), Erlotinib (aka, Tarceva, CP358, OSI-774, or NSC718781), AG494 and AG1478 were purchased from Cayman Chemical (Ann Arbor, MI, USA) and EMD Chemicals (Gibbstown, NJ, USA). Unless indicated otherwise, all chemicals were purchased from Sigma-Aldrich (St. Louis, MO, USA) or Fisher Scientific (Pittsburgh, PA, USA).

### Recombinant adenoviruses expressing BMP9, EGF, RFP and GFP

Recombinant adenoviruses were generated using AdEasy technology as described [13, 14, 25, 37, 38]. The coding regions of human BMP9 and EGF were PCR amplified and cloned into an adenoviral shuttle vector and subsequently used to generate recombinant adenoviruses in HEK293 cells. The resulting adenoviruses were designated as AdBMP9 and AdEGF. AdBMP9 also expresses GFP, whereas AdEGF expresses RFP as a marker for monitoring infection efficiency. Analogous adenovirus expressing only monomeric RFP (AdRFP) or GFP (AdGFP) was used as controls [18, 19, 37–45].

### RNA isolation and semi-quantitative RT-PCR

Total RNA was isolated using TRIzol RNA Isolation Reagents (Invitrogen, Grand Island, NY, USA) and used to generate cDNA templates by RT reaction with hexamer and M-MuLV Reverse Transcriptase (New England Biolabs, Ipswich, MA, USA). The cDNA products were diluted 5- to 10-fold and used as PCR templates. Semi-quantitative RT-PCR was carried out as described [19, 21, 25, 41, 43, 46–48]. PCR primers (Table S1) were designed using the Primer3 program to amplify the genes of interest (∼150–180 bp). A touchdown cycling program was as follows: 94°C for 2 min. for one cycle; 92°C for 20 sec., 68°C for 30 sec. and 72°C for 12 cycles decreasing 1°C per cycle; and then at 92°C for 20 sec., 57°C for 30 sec. and 72°C for 20 sec. for 23–28 cycles, depending on the abundance of a given gene. PCR products were resolved on 1.5% agarose gels. All samples were normalized by the expression level of GAPDH.

### Alkaline phosphatase (ALP) assay

Alkaline phosphatase activity was quantitatively assessed using a modified Great Escape SEAP Chemiluminescence assay (BD Clontech, Mountain View, CA, USA) and/or histochemical staining assay (using a mixture of 0.1 mg/ml napthol AS-MX phosphate and 0.6 mg/ml Fast Blue BB salt) as described [13, 14, 16–19, 25, 36, 41, 42, 45]. For the chemiluminescence assays, each assay condition was performed in triplicate and the results were repeated in at least three independent experiments. Alkaline phosphatase activity was normalized by total cellular protein concentrations among the samples (using the BCA protein assay).

### Alizarin Red S staining

C3H10T1/2 and iMEF cells were seeded in 12- or 24-well cell culture plates and infected with AdBMP9, AdEGF, AdGFP or AdBMP9+AdEGF. Infected cells were cultured in the presence of ascorbic acid (50 μg/ml) and β-glycerophosphate (10 mM). At 14 days after infection, mineralized matrix nodules were stained for calcium precipitation by means of Alizarin Red S staining, as described previously [13, 14, 16–19, 25, 36, 41]. Cells were fixed with 0.05% (v/v) glutaraldehyde at room temperature for 10 min. After being washed with distilled water, fixed cells were incubated with 0.4% Alizarin Red S (Sigma-Aldrich) for 5 min., followed by extensive washing with distilled water. The staining of calcium mineral deposits was recorded under bright field microscopy.

### Foetal limb explant culture

The forelimbs (*i.e*. humerus containing elbow joint) of mouse embryos (E18.5) were dissected under sterile conditions and incubated in DMEM (Invitrogen) containing 0.5% bovine serum albumin, 50 μg/ml ascorbic acid, 1 mM β-glycerophosphate and 100 μg/ml penicillin–streptomycin solution at 37°C in humidified air with 5% CO_2_ for up to 14 days as described [21, 42, 45]. Limb explants were directly infected with AdGFP or AdBMP9 24 hrs after dissection in the presence of EGF and/or Gefitinib. The medium was changed every 2–3 days. Cultured tissues were observed in different time-points under microscope to confirm the survival of cells and the expression of fluorescence markers. New bone formation was visualized by adding calcein (100 mM) to the medium.

### Immunohistochemical staining

Cultured cells were treated, fixed with 10% formalin and washed with PBS. The fixed cells were permeabilized with 1% NP-40 and blocked with 10% goat serum, followed by incubation with an anti-osteocalcin (OCN), or osteopontin (OPN) antibody (Santa Cruz Biotechnology, Santa Cruz, CA, USA) for 1 hr. After being washed, cells were incubated with biotin-labelled secondary antibody for 30 min., followed by incubating cells with streptavidin–HRP conjugate for 20 min. at room temperature. The presence of the expected protein was visualized by 3,3′-Diaminobenzidine (DAB) staining and examined under a microscope. Stains with control IgG were used as negative controls.

### Stem cell implantation and μCT analysis

Subconfluent iMEFs were infected with AdBMP9/AdRFP or AdBMP9/AdEGF. At 16 hrs after infection, cells were harvested, and resuspended in PBS for subcutaneous injection (5 × 10^6^/injection) into the flanks of athymic nude (nu/nu) mice (five animals per group, 4–6 week old, female, Harlan Sprague Dawley, Indianapolis, IN, USA). At 4 weeks after implantation, animals were killed. The retrieved specimens were fixed and imaged using the μCT component of a GE Triumph (GE Healthcare, Piscataway, NJ, USA) trimodality preclinical imaging system. All image data analysis was performed with Amira 5.3 (Visage Imaging Inc., San Diego, CA, USA), and 3D volumetric data and bone mean density heat maps were obtained as previously described [21, 28, 35, 42, 45].

### Haematoxylin and eosin, trichrome and alcian blue staining

The retrieved tissues were fixed, decalcified in Cal-Ex II Fixative/Decalcifier (Fisher Scientific) overnight and embedded in paraffin. Serial sections of the embedded specimens were stained with haematoxylin and eosin. Masson Trichrome and Alcian Blue stains were performed as previously described [14, 17–19, 25, 28, 36, 41, 42, 45].

### ChIP analysis

Subconfluent iMEFs were infected with AdGFP or AdBMP9. At 30 hrs after infection, cells were cross-linked and subjected to ChIP analysis as previously described [18, 19, 41]. Smad1/5/8 antibody (Santa Cruz Biotechnology) or control IgG was used to pull down the protein-DNA complexes. The presence of *Egfr* promoter sequence was detected by PCR amplification using two pairs of primers corresponding to mouse *Egfr* promoter region.

### Statistical analysis

All quantitative experiments were performed in triplicate and/or repeated three times. Data were expressed as mean ± SD. Statistical significances between vehicle treatments versus drug treatments were determined by one-way anova and the Student's *t-*test. A value of *P* < 0.05 was considered statistically significant.

## Results

### EGF enhances BMP9-induced osteogenic differentiation of MSCs

**Fig. 1:**
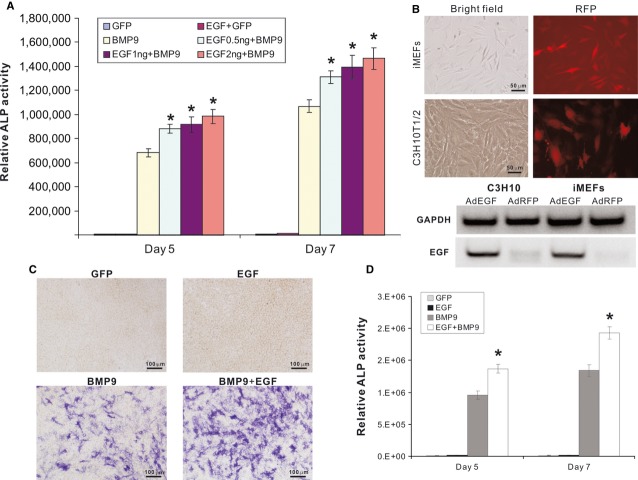
Epidermal growth factor (EGF) synergizes with BMP9 in inducing early osteogenic marker alkaline phosphatase (ALP) activity in mesenchymal stem cells (MSCs). (**A**) EGF augments BMP9-induced ALP activity in a dose-dependent fashion. Subconfluent iMEFs were infected with AdBMP9 or AdGFP in the presence of varied concentrations of EGF (0–2.0 ng/ml). At days 5 and 7, cells were lysed and subjected to ALP assays. Each assay conditions were carried out in triplicate. ‘*’ indicates *P* < 0.05 when compared with the BMP9 alone group. (**B**) AdEGF can effectively infect MSCs and express the transgene. Subconfluent C3H10T1/2 and iMEF cells were infected with AdEGF virus for 36 hrs; and the transduction efficacy were recorded under a fluorescence microscope (RFP). AdEGF-mediated human EGF expression was analysed by semi-quantitative RT-PCR using PCR primers located within the coding region of human EGF. (**C** and **D**) Exogenous expression of EGF enhances BMP9-induced ALP activity in MSCs. Subconfluent iMEFs were infected with equal titre of AdBMP9, AdEGF, AdGFP or AdBMP9/AdEGF. Cells were fixed for ALP histochemical staining assays (**C**) at day 7, or collected and subjected to ALP quantitative bioluminescence assays (**D**) at day 5 or day 7. Each assay conditions were carried out in triplicate. ‘*’ indicates *P* < 0.05 when compared with the BMP9 alone group. See Methods for details.

**Fig. 2:**
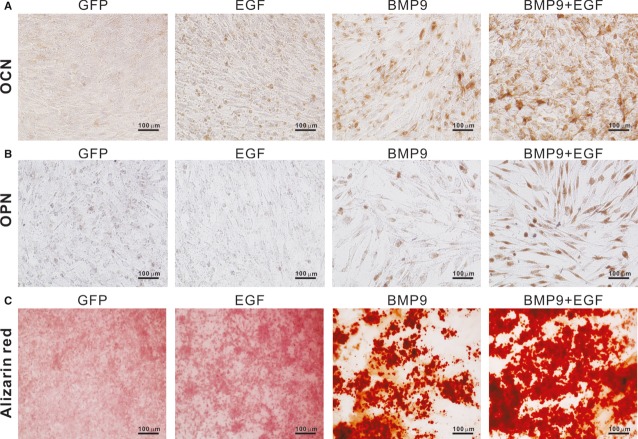
Epidermal growth factor (EGF) enhances BMP9-induced late osteogenic markers and mineralization in mesenchymal stem cells. (**A** and **B**) EGF promotes BMP9-induced late osteogenic markers. Subconfluent iMEFs were infected with adenovirus(es) expressing the indicated transgenes. At 14 days after infection, cells were fixed and subjected to immunohistochemical staining with a primary antibody against osteocalcin (OCN) (**A**) or osteopontin (OPN) (**B**). (**C**) EGF augments BMP9-induced matrix mineralization. Subconfluent iMEFs were infected with adenovirus(es) expressing the indicated transgenes and cultured in mineralization medium for 14 days. Cells were fixed and subjected to Alizarin Red S staining. Each assay condition was repeated in at least three independent experiments. Representative results are shown. See Methods for details.

We previously identified that BMP9 is one of the most osteogenic BMPs in MSCs [8, 13, 14, 19, 20]. Although we have identified several early targets that are important mediators of BMP9-induced osteogenic signalling [15–18, 20, 21], much remain to be learnt about how BMP9 cross-talks with other signalling pathways. Here, we investigated the possible cross-talk between BMP9 and EGF signalling pathways. Using a well-characterized MSC iMEFs, we found that rhEGF enhanced BMP9-induced early osteogenic marker ALP activity in a dose-dependent fashion, although rhEGF alone did not induce any detectable ALP activity (Fig. 1A). To further confirm these results, we constructed a recombinant adenovirus that expresses human EGF and demonstrated that this vector was able to transduce MSCs, such as iMEFs and C3H10T1/2 with high efficiency (Fig. 1B). Co-expression of BMP9 and EGF in MSCs led to a significant increase in ALP activity qualitatively (Fig. 1C) and quantitatively (Fig. 1D). Furthermore, exogenous expression of EGF was shown to enhance BMP9-induced expression of the late osteogenic markers osteocalcin (OCN) (Fig. 2A) and OPN (Fig. 2B). BMP9-induced matrix mineralization was significantly enhanced by co-expression of EGF in MSCs (Fig. 2C). These *in vitro* results strongly suggest that EGF signalling may synergize with BMP9-induced osteogenic signalling, although EGF signalling alone is seemingly insufficient to initiate osteogenic differentiation of MSCs.

### BMP9-induced osteogenic differentiation can be effectively blocked by EGFR inhibitors

**Fig. 3:**
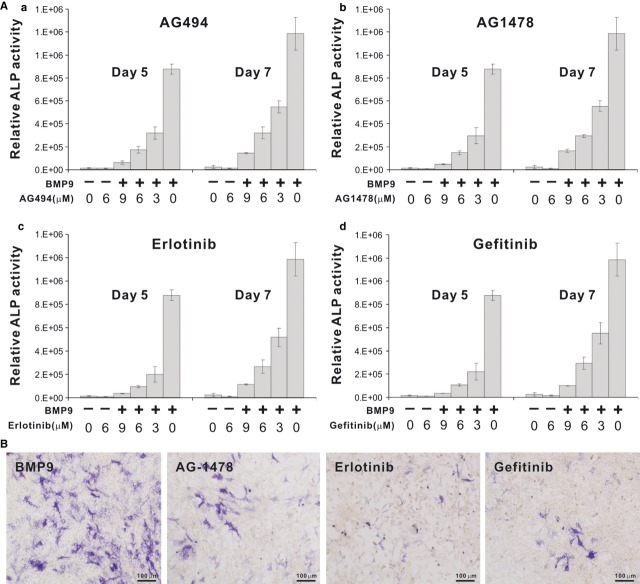
Epidermal growth factor receptor (EGFR) tyrosine kinase inhibitors can blunt BMP9-induced osteogenic differentiation of mesenchymal stem cells (MSCs). (**A**) EGFR tyrosine kinase inhibitors effectively block BMP9-induced alkaline phosphatase (ALP) activity in MSCs in a dose-dependent fashion. Subconfluent iMEF cells were infected with AdBMP9 or AdGFP and treated with varied concentrations of AG494 (a), AG1478 (b), Erlotinib (c) and Gefitinib (d). ALP activity was quantitatively determined at days 5 and 7 respectively. (**B**) Similar experiments were set up as in (**A**). All inhibitors were used at 6 μM. ALP activity was qualitatively assessed at day 7 using histochemical staining. Each assay condition was carried out in triplicate. Representative results are shown.

Epidermal growth factor initiates downstream events by activating its tyrosine kinase receptor EGFR (aka HER1). We sought to determine if commonly EGFR inhibitors would exert any effect on BMP9-induced osteogenic differentiation of MSCs. We tested four EGFR inhibitors, AG494, AG1478, Erlotinib and Gefitinib, and found all of them inhibited BMP9-induced ALP activity in a dose-dependent manner (Fig. 3A). Among the four tested inhibitors, Erlotinib and Gefitinib are clinically used as anticancer drugs, which were shown to be slightly more effective than AG494 and AG1478 in terms of inhibiting BMP9-induced ALP activity (Fig. 3A and B). Thus, these *in vitro* findings suggest that EGFR signalling may play an important role in BMP9-regulated osteogenic differentiation, although these inhibitors may also target other tyrosine kinase.

### BMP9-regulated osteogenesis of mouse foetal limbs is promoted by EGF, but inhibited by EGFR inhibitors

**Fig. 4:**
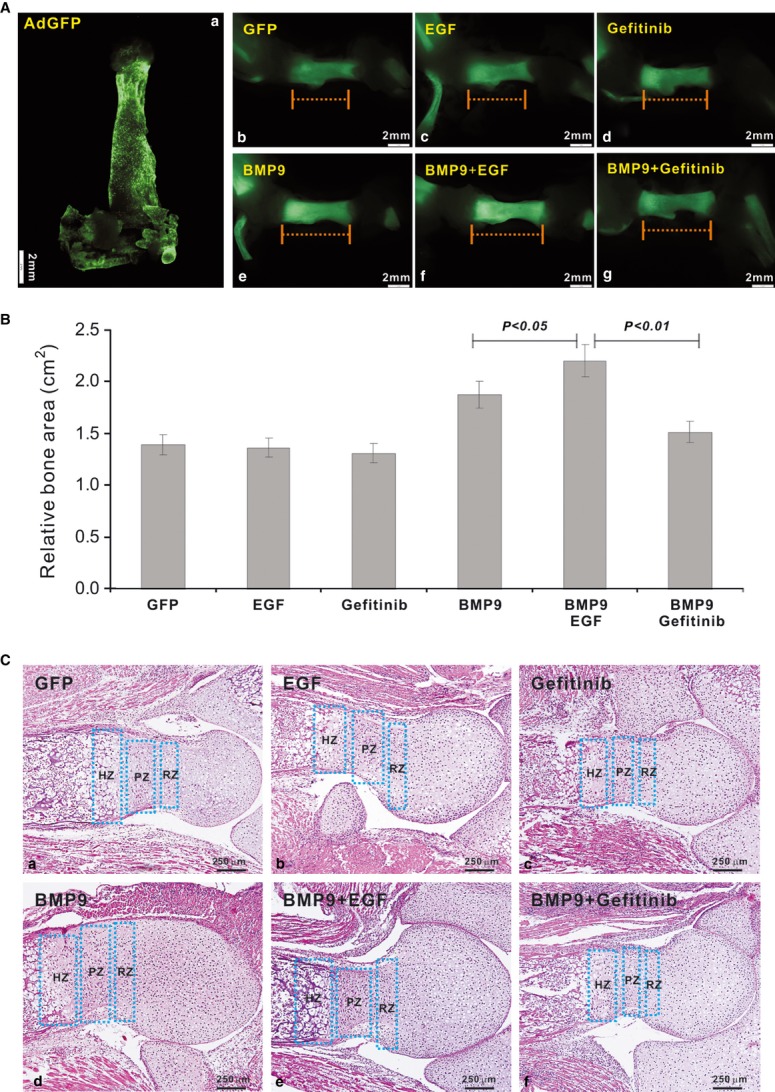
EGF and BMP9 act synergistically in promoting osteogenesis in mouse foetal limb explant culture. (**A**) Mouse humerus containing elbow joint was isolated from E18.5 embryos and cultured in BSA medium. The cultured explants (*n* = 8 per assay condition) were infected with AdBMP9 or AdRFP in the presence of rhEGF (2 ng/ml) or Gefitinib (6 μM), and cultured for up to 14 days, with medium changed every 2–3 days. At day 12, calcein was added to the culture (see Methods). The tissues were harvested on day 14 and examined under a stereo fluorescence microscope (b-g). (**B**) The fluorescence-positive area (in cm^2^) for each sample was measured and analysed using NIH ImageJ. (**C**) Haematoxylin and eosin staining. The harvested tissues were fixed, paraffin-embedded and subjected to haematoxylin and eosin staining. Representative images are shown. RZ, resting zine; PZ, proliferating zone; HZ, hypertrophic zone.

We sought to analyse the effect of EGF and BMP9 on developing bone using previously described *ex vivo* foetal limb culture assay [21, 42]. We isolated mouse humerus containing elbow joint (with some soft tissues attached) from E18.5 embryos, which could be effectively transduced by adenoviral vectors (Fig. 4A*, panel a*). The isolated limbs were infected with AdBMP9 or AdGFP with or without EGF or Gefitinib. New bone formation was visualized by the incorporation of fluorescent dye calcein (Figure S1A). We found that the BMP9 group and BMP9+EGF group exhibited the highest fluorescence intensity, indicating active new bone formation (Fig. 4A*, panels b–g*). The BMP9+EGF group exhibited the largest fluorescence-positive area and was higher than that of BMP9 alone group (*P* < 0.05), while Gefitinib significantly inhibited BMP9-promoted bone formation (*P* < 0.01; Fig. 4B). Consistent with the *in vitro* results, EGF stimulation alone did not significantly promote osteogenesis, whereas Gefitinib might slightly inhibit osteogenesis, but the effect was not statistically significant (Fig. 4B). These results were further confirmed by histological analysis. The height and different chondrocyte zones at humerus growth plate did not exhibit any significant differences in EGF or Gefitinib treatment group compared with that of GFP control (Fig. 4C*, panels a versus b & c*). However, BMP9 or BMP9+EGF treatment led to a significant expansion of the humerus growth plate and endochondral bone formation (Fig. 4C*, panels d & e*). Notably, a combination of BMP9 and EGF led to a marked increase in cell numbers at the proliferating zone (Fig. 4C*, panel e*, Figure S1B). Conversely, the BMP9-induced growth plate expansion was blunted by EGFR inhibitor Gefitinib (Fig. 4C*, panels d versus f*). These *in vitro* results suggest that EGF may potentiate BMP9-induced endochondral ossification by increasing proliferative chondrocytes at the growth plate.

### EGF enhances BMP9-induced ectopic bone formation and matrix maturation in stem cell implantation assay

**Fig. 5:**
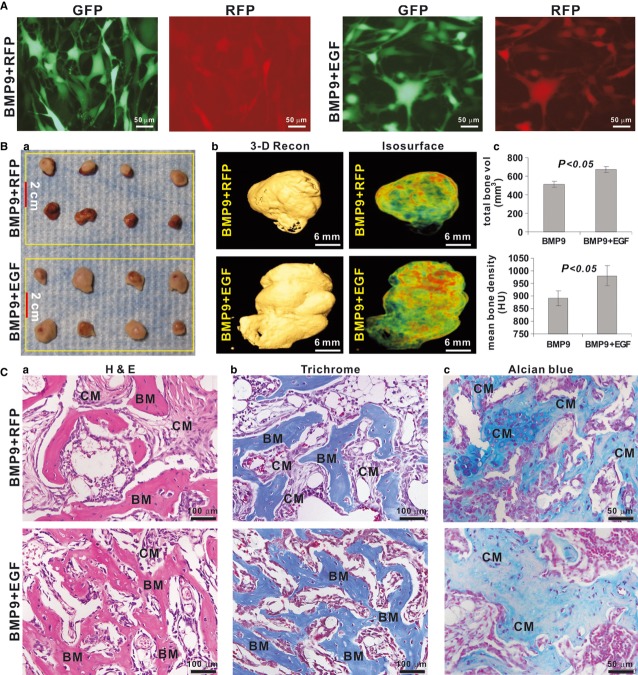
Epidermal growth factor (EGF) accelerates BMP9-induced bone formation and matrix maturation in stem cell implantation assays. Subconfluent iMEF cells were co-infected with AdBMP9, AdRFP and/or AdEGF for 16 hrs (**A**) and harvested to inject into athymic mice subcutaneously. Bony masses were found in BMP9 + RFP and BMP9 + EGF transduced cell groups at 4 weeks (**B**, panel a). No masses of any kind were formed in the cells transduced with AdRFP or AdEGF alone. The retrieved masses were fixed in formalin and subjected to micro-CT scanning. The scanning data were analysed with Amira 5.3 software to obtain 3D reconstruction and isosurface images (**B**, panel b), isosurface volume rendering (bone mass) and relative bone mean density (in Hounsfield unit, HU) (**B**, panel c). Red indicates higher HU, whereas green indicates the lower HU. (C) Histological analysis. The retrieved ectopic bone masses were fixed, decalcified and subjected to haematoxylin and eosin staining (a), trichrome staining (b) and alcian blue staining (c). BM, bone matrix; CM, chondroid matrix. Representative images are shown.

We further carried out stem cell implantation assays to determine the EGF effect on BMP9-induced bone formation *in vivo*. Subconfluent iMEFs were effectively co-infected with BMP9, RFP and/or EGF for 16 hrs (Fig. 5A). The infected cells were harvested to inject into athymic mice subcutaneously. Bony masses were found in BMP9+RFP and BMP9+EGF (Fig. 5B*, panel a*)-transduced cell groups at 4 weeks. No masses were formed in the cells transduced with RFP or EGF alone. The gross masses retrieved from BMP9+EGF group were larger than that from the BMP9 only group, which was confirmed by μCT scanning (Fig. 5B*, panels a & b*). The 3D isosurface analysis of the μCT scanning data revealed that the total bone volume in BMP9+EGF group was larger than that of BMP9 only group (Fig. 5B*, panel c*). Furthermore, a combination treatment of BMP9 and EGF promoted the mineralization of bone masses as demonstrated by μCT analysis of relative bone mean density in Hounsfield unit (Fig. 5B*, panel c*). Histological analysis of the retrieved ectopic bone masses indicated that MSCs treated with both BMP9 and EGF led to higher numbers of trabeculae and more mature bone matrix than that of the BMP9 alone group (Fig. 5C*, panel a*). These results were further confirmed by Masson's Trichrome staining of the retrieved bone masses, as more mature osteoid matrices were found in the specimens treated with both BMP9 and EGF (Fig. 5C*, panel b*). Conversely, alcian blue staining indicated that iMEFs co-infected with AdBMP9 and AdEGF exhibited slightly less cartilage matrix staining than those infected with AdBMP9 only (Fig. 5C*, panel c*). Thus, these *in vivo* results demonstrate that EGF can accelerate BMP9-induced osteoid matrix mineralization.

### BMP9 cross-talks with EGF signalling pathway in regulating osteogenic differentiation of MSCs

**Fig. 6:**
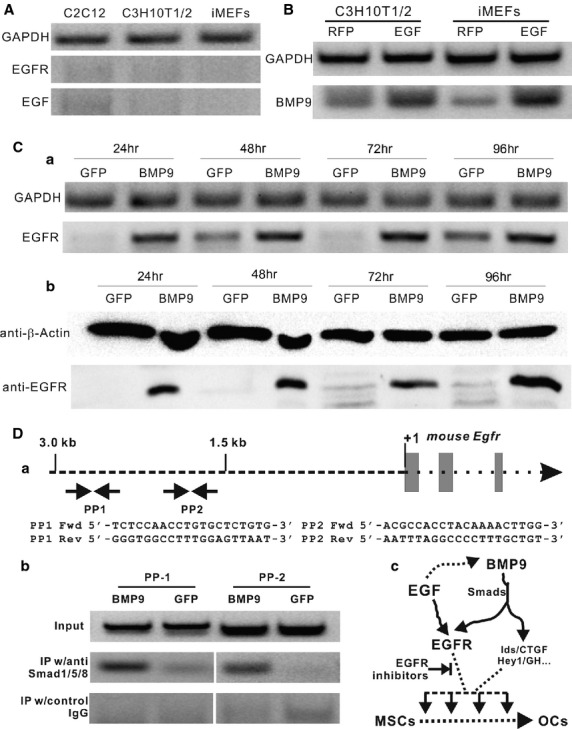
Cross-talk between epidermal growth factor (EGF) and BMP9 signalling pathways in mesenchymal stem cells (MSCs). (**A**) Endogenous expression of EGF and epidermal growth factor receptor (EGFR) in pre-osteoblast progenitor cells. Subconfluent C3H10T1/2, iMEFs and C2C12 cells were cultured under 1% FBS condition for 24 hrs. Total RNA was collected and subjected to RT-PCR analysis using primers specific for mouse EGF and EGFR. GAPDH was used as an internal normalization standard. (**B**) EGF stimulates BMP9 expression in MSCs. Subconfluent C3H10T1/2 and iMEF cells were infected with AdEGF or AdRFP for 30 hrs. Total RNA was collected for RT-PCR analysis using primers specific for mouse BMP9. GAPDH was used as an internal normalization standard. (**C**) BMP9 up-regulates EGFR expression in MSCs. Subconfluent iMEFs were infected with AdBMP9 or AdGFP. Total RNA was isolated at the indicated time-points and subjected to RT-PCR analysis using primers specific for mouse EGFR, whereas GAPDH was used as an internal normalization standard (a). For Western blotting analysis, the total cell lysate was subjected to SDS-PAGE and probed with a primary antibody against EGFR, whereas β-actin was used as a loading control (b). (**D**) BMP9 regulates EGFR expression through Smad signalling in MSCs. (*a*) Schematic depiction of mouse *Egfr* promoter region. The approximate locations of the two pairs of primers, PP-1 and PP-2, were indicated. ‘+1’ denotes the start position of exon 1 of mouse EGFR. (*b*) ChIP analysis. Subconfluent iMEF cells were infected with AdGFP or AdBMP9 for 30 hrs. Cells were cross-linked. Genomic DNA was sonicated, following immunoprecipitation with Smad1/5/8 antibody or IgG. The retrieved genomic DNA was subjected to PCR using the two pairs of primers PP-1 and PP-2. The arrows indicate the locations of the expected products. Control assays that demonstrated that a similar amount of input materials was used for immunoprecipitation experiments. ChIP analysis was performed in three independent experiments, and the representative results are shown. (*c*) A mode of action. Upon BMP9 stimulation of MSCs, several important downstream targets including EGFR are up-regulated. EGF signalling synergizes with other BMP9 targets and leads to efficient osteogenesis. Intriguingly, EGF itself may up-regulate BMP9 expression *via* a yet-to-be-determined mechanism. EGFR inhibitors can significantly block BMP9-induced osteogenic signalling. Ids, inhibitors of DNA-binding factors; CTGF, connective tissue growth factor; GH, growth hormone; OCs, osteocytes.

Our *in vitro* and *in vivo* results show that EGF can significantly enhance BMP9-induced osteogenic differentiation of MSCs, suggesting that EGF may function in parallel and/or upstream of BMP9 signalling. The fact that EGFR inhibitors can block BMP9-induced osteogenic activity indicates that BMP9 could act upstream of EGFR function. We sought to determine the mechanistic aspects of BMP9 and EGF signalling in MSCs. Under unstimulated conditions, the pre-osteoblast progenitors (such as C3H10T1/2, iMEFs and C2C12 cells) expressed very low or undetectable levels of EGF and EGFR (Fig. 6A). When MSC cells (*e.g*. C3H10T1/2 and iMEFs) were stimulated with EGF, we found that BMP9 expression was apparently up-regulated (Fig. 6B), suggesting that EGF may act at least in part upstream of BMP9 signalling in MSCs. Consistent with this possibility, EGF was shown to enhance BMP9-induced phosphorylation of Smad1/5/8 (Figure S2). Furthermore, BMP9 stimulation in MSCs led to a significant increase in EGFR expression both at mRNA level (Fig. 6C*, panel a*) and protein level (Fig. 6C*, panel b*), while we did not observed any detectable changes in EGF expression (data not shown).

These results suggest that EGFR may be directly regulated by BMP9 signalling. We sought to determine if BMP9 regulates EGFR expression through Smad pathway using the chromatin immunoprecipitation (ChIP) assay. We analysed the possible presence of BMP R-Smad binding site(s) in the 3.0 kb *Egfr* promoter region using two pairs of PCR primers (Fig. 6D, *panel a*). For ChIP analysis, iMEFs were infected with AdGFP or AdBMP9 and cross-linked. Genomic DNA–protein complexes were sonicated and immunoprecipitated with anti-Smad1/5/8 antibody. The retrieved genomic DNA was subjected to PCR amplification using the two pairs of primers, PP1 and PP2. Although the genomic DNA input was comparable among the samples, primer pairs PP1 and PP2 were shown to amplify the expected fragments in BMP9-stimulated cells, but not in GFP control cells or in the samples precipitated by the control IgG (Fig. 6D, *panel b*). Thus, these results strongly suggest that EGF may be a direct downstream target of BMP9 signalling in MSCs.

Taken together, our results suggest a following mode of action about the cross-talk between BMP9 and EGF signalling pathways. Upon BMP9 stimulation of MSCs, several important downstream targets including EGFR are up-regulated. Epidermal growth factor signalling synergizes with other BMP9 targets and leads to efficient osteogenesis. BMP9-induced osteogenic signalling can be blocked by EGFR inhibitors. Intriguingly, EGF itself may up-regulate the expression of BMP9 through a yet-to-be-determined mechanism (Fig. 6D*, panel c*).

## Discussion

We previously identified BMP9 as one of the most osteogenic BMPs both *in vitro* and *in vivo* [8, 13–20]. As one of the least studied BMPs, BMP9 may exert its signalling activity by regulating a distinct set of downstream mediators in MSCs. We have recently identified several downstream targets in MSCs upon BMP9 stimulation [8, 15–18, 20]. As for any major signalling molecules, BMP9 may fulfil its biological functions by cross-talking with other pathways. In this study, we show that EGF potentiates BMP9-induced early and late osteogenic markers of MSCs, which can be effectively inhibited by EGFR inhibitors Gefitinib and Erlotinib or protein tyrosine kinase inhibitors AG-1478 and AG-494. Epidermal growth factor significantly augments BMP9-induced endochondral bone formation in the cultured mouse foetal limb explants, which can be blocked by EGFR inhibitors. *In vivo* stem cell implantation experiments reveal that exogenous expression of EGF in MSCs effectively potentiates BMP9-induced ectopic bone formation, yielding larger and more mature trabecular bone masses. Mechanistically, EGF is shown to induce BMP9 expression in MSCs, whereas EGFR expression is directly up-regulated by BMP9 through Smad1/5/8 signalling pathway. Thus, the regulatory circuitry of EGF and BMP9 signalling pathways in MSCs may underline their important roles in regulating osteogenic differentiation.

Our results indicate that EGF itself exhibits negligible capability to induce osteogenic differentiation of MSCs. In fact, it was reported that the activation of EGF signalling may promote the proliferation and expansion of the pre-osteoblast progenitor cells at the expense of lineage-specific differentiation [30, 49, 50]. However, it was also shown that EGF stimulation enhances osteogenic differentiation of human MSCs in the presence of chemical cues [51]. Although no satisfactory explanation is offered about the conflicting results, one possibility is that the EGF effect on MSC cells may be dependent on the proliferation *versus* differentiation status of the pre-osteoblast progenitor cells. Another possible explanation is that the biological outcome of the EGF-stimulated MSCs may be significantly affected by the potency/efficacy of the differentiation cue. We have demonstrated that osteogenic BMPs (including BMP9)–induced osteogenesis is a well-coordinated process of the proliferative expansion and terminal differentiation of pre-osteoblast progenitor cells [4, 8, 16, 20, 52]. Our previous studies have demonstrated that BMP9 is one of the most osteogenic factors [4, 8, 13, 14, 20]. Thus, our current studies indicate that the EGF-stimulated expansion of progenitor cells can be efficiently driven to osteogenic lineage and terminal differentiation by BMP9. Consistent with our findings is an earlier study, which showed that overexpression of *Egf* in transgenic mice using the β-actin promoter results in growth retardation and over-proliferation of osteoblasts [53].

It has been reported that EGFR signalling plays an important role in growth plate development, endochondral bone formation and longitudinal bone growth [29, 31, 54]. The EGFR family of receptor tyrosine kinases includes EGFR/ErbB1, HER2/ErbB2, HER3/ErbB3 and HER4/ErbB [31, 55]. Epidermal growth factor receptor binds several ligands including EGF, TGF-α, β-cellulin, epiregulin and amphiregulin. Upon ligand binding, EGFR dimerizes and undergoes auto- or transphosphorylation on tyrosine residues in the intracellular domain, leading to the activation of several important intracellular signalling pathways, such as Ras-Raf-MAP-kinase and PI-3 Kinase-Akt. ErbB2 is the preferred partner for EGFR, and the signals mediated by EGFR/ErbB2 account for most of EGFR's biological activities [31, 55]. *Egfr*-deficient mice exhibit impaired endochondral ossification, probably secondary to a defect in hypertrophic chondrocyte maturation and osteoblastic cell proliferation, and delayed primary endochondral ossification because of defective osteoclast recruitment [54, 56]. More recently, it was shown that *in vivo* administration of Gefitinib or mice with cartilage-specific *Egfr* inactivation leads to defective endochondral ossification and hypertrophic cartilage enlargement as a result of suppressed osteoclastogenesis [57], although it is not clear if these effects resulted from the blockade of TGFα signalling. Nonetheless, it has been shown that the Egfr dominant–negative mutant mice exhibited a remarkable decrease in tibial trabecular bone mass with abnormalities in trabecular number and thickness, whereas mice with a constitutively active Egfr allele displayed increases in trabecular and cortical bone content [58]. Thus, our findings are supported by these prior studies on EGFR functions in bone and skeletal development.

Finally, it is noteworthy that aberrant activations of EGFR signalling are frequently observed in many types of advanced stage cancers and bone metastases [59, 60]. Various EGFR tyrosine kinase inhibitors, such as Gefitinib and Erlotinib, have shown promising results in clinical and/or preclinical anticancer studies [59–61]. Further investigations may be required to determine if the use of the EGFR inhibitors could affect the bone density in cancer patients, leading to pathological fractures. Furthermore, it is also important to investigate if the use of these inhibitors to treat bone metastases could worsen osteolytic lesions.

In summary, we have demonstrated that EGF potentiates BMP9-induced early and late osteogenic markers of MSCs, which can be effectively blunted by EGFR inhibitors. EGF also augments BMP9-induced endochondral bone formation in the cultured mouse foetal limb explants. Exogenous expression of EGF in MSCs effectively potentiates BMP9-induced ectopic bone formation. Mechanistically, EGF is shown to induce BMP9 expression in MSCs, whereas EGFR expression is directly up-regulated by BMP9 through Smad1/5/8 signalling pathway. Thus, the cross-talk between EGF and BMP9 pathways in MSCs may play an important role in regulating osteogenic differentiation. Harnessing the synergy between BMP9 and EGF should be beneficial for enhancing osteogenesis in regenerative medicine.

## Supporting information

**Figure S1**. (**A**) Different fluorescence signals detected in adenovirus-transduced soft tissues (a) *versus* calcein-stained new bone formation (b). (**B**) Immunohistochemical staining analysis indicates that the humerus growth plate from BMP9+EGF-treated limb explants exhibited higher PCNA-positive cells than that of the BMP9-treated alone (63% *versus* 32%).

**Figure S2**. EGF enhances BMP9-induced phosphorylation of Smad1/5/8. Subconfluent iMEFs were infected with GFP, BMP9, EGF and BMP9+EGF adenoviruses. At 24 hrs after infection, cells were fixed and immunostained with a p-Smad1/5/8 antibody (Santa Cruz Biotechnology). Positively stained cells were indicated by arrows. Representative results are shown.

**Table S1.** Oligonucleotides used for cloning, RT-PCR and ChIP.
