# Supplementary figures and images for: Cross-talk between EGF and BMP9 signalling pathways regulates the osteogenic differentiation of mesenchymal stem cells
Source: J Cell Mol Med. 2013 Jul 11;17(9):1160–72. doi: 10.1111/jcmm.12097 (PMC4118175; doi:10.1111/jcmm.12097)

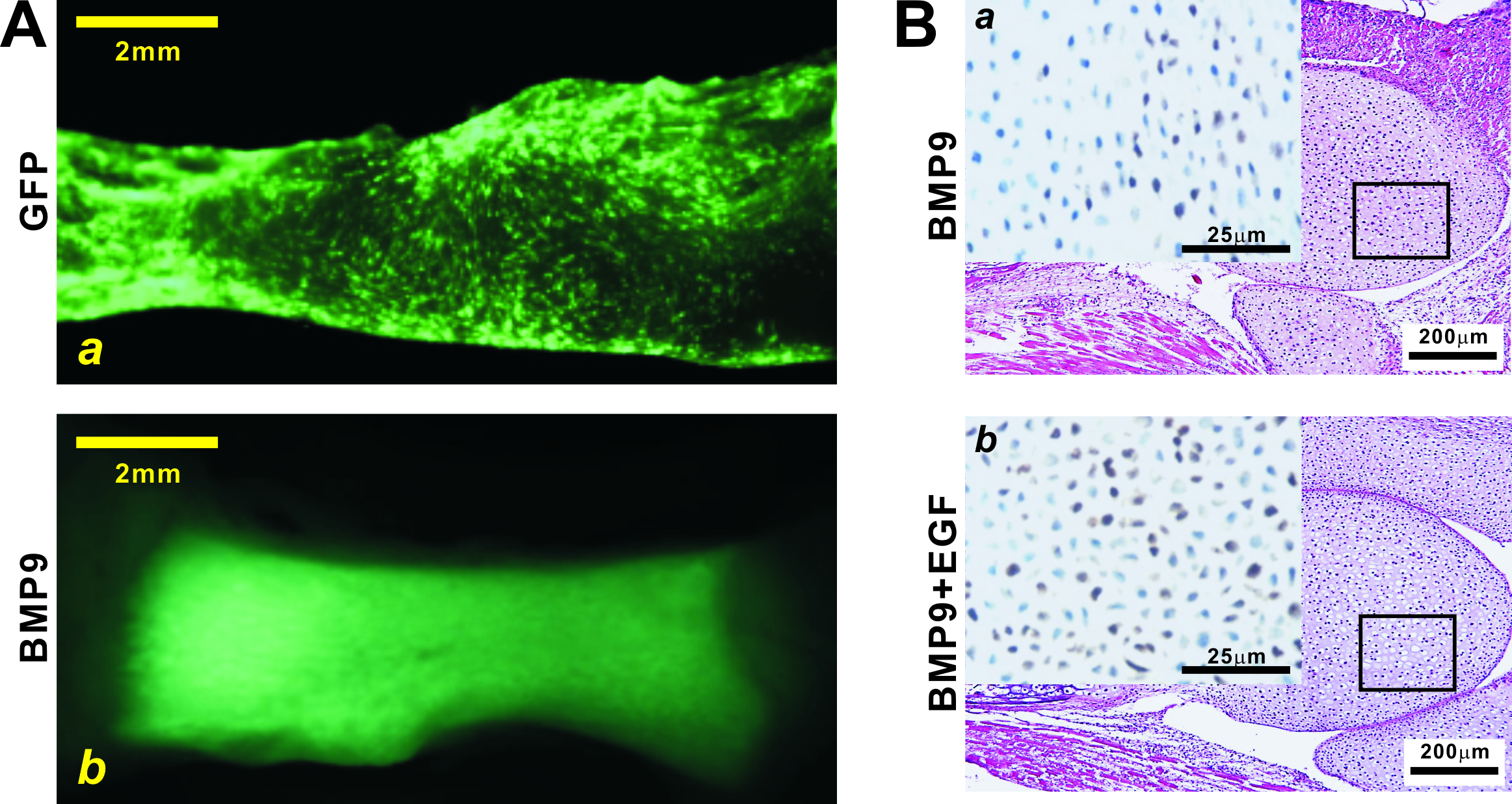

Supplement: Supplementary file 1 [file jcmm0017-1160-SD1.tif]

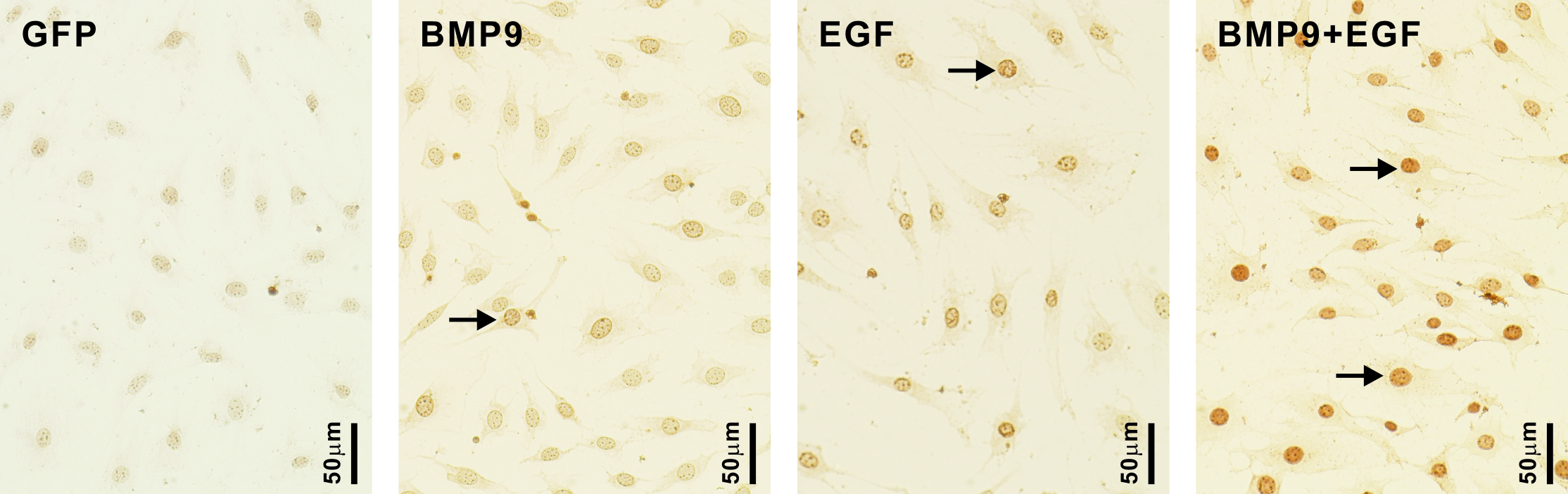

Supplement: Supplementary file 2 [file jcmm0017-1160-SD2.tif]
